# Design and fabrication of superhydrophobic cellulose nanocrystal films by combination of self-assembly and organocatalysis

**DOI:** 10.1038/s41598-023-29905-1

**Published:** 2023-02-23

**Authors:** Rana Alimohammadzadeh, Italo Sanhueza, Armando Córdova

**Affiliations:** Department of Natural Science and Technology, Mid Sweden University, Holmgatan 10, 851 70 Sundsvall, Sweden

**Keywords:** Chemistry, Materials science, Nanoscience and technology

## Abstract

Cellulose nanocrystals, which have unique properties of high aspect ratio, high surface area, high mechanical strength, and a liquid crystalline nature, constitute a renewable nanomaterial with great potential for several uses (e.g., composites, films and barriers). However, their intrinsic hydrophilicity results in materials that are moisture sensitive and exhibit poor water stability. This limits their use and competitiveness as a sustainable alternative against fossil-based materials/plastics in packaging, food storage, construction and materials application, which cause contamination in our oceans and environment. To make cellulose nanocrystal films superhydrophobic, toxic chemicals such as fluorocarbons are typically attached to their surfaces. Hence, there is a pressing need for environmentally friendly alternatives for their modification and acquiring this important surface property. Herein, we describe the novel creation of superhydrophobic, fluorocarbon-free and transparent cellulose nanocrystal films with functional groups by a bioinspired combination of self-assembly and organocatalytic surface modification at the nanoscale using food approved organic acid catalysts. The resulting film-surface is superhydrophobic (water contact angle > 150°) and has self-cleaning properties (the lotus effect). In addition, the superhydrophobic cellulose nanocrystal films have excellent water stability and significantly decreased oxygen permeability at high relative humidity with oxygen transmission rates better than those of commonly used plastics.

## Introduction

The introduction of superhydrophobic surfaces into a variety of materials so that they exhibit nonwetting properties has inspired researchers both in academia and industry^1–9^. Several applications have been investigated in fields such as packaging, biomedical applications, defense, engineering, sensors, green electronics, apparel and aerospace^1–10^. The surface texture or chemical properties of superhydrophobic surfaces generally contribute to their water repellency^1–10^. In nature, the most famous superhydrophobic surface is the leaves of lotus plants (e.g., *Nelumbo nucifera*), which have a high contact angle (~ 160°) and self-cleaning properties (the lotus effect)^11,12^. This observation is due to a combination of hydrophobic properties and nano- and microstructural roughness provided by hydrophobic epicuticular waxes (wax crystals) and the papillae formed by the epidermal (outermost) cells of the lotus leaf, respectively^11,12^. Mimicking these characteristics found in nature allow for accomplishing superhydrophobicity on various material surfaces^1–10^.

Superhydrophobic properties are characterized by a water contact angle (WCA) > 150° and various adhesion levels of water on the surface determined by dynamic contact angle measurements^4^. The hydrophobic properties of the surface are highly dependent on the topology and morphology of surfaces, as characterized by the Wenzel and Cassie–Baxter equations^1,4,13^. In the Wenzel state, the water droplet is in full contact with the surface, and the Young’s contact angle for a smooth surface is amplified by a roughness parameter^4^. In the Cassie-Baxter state, the liquid does not penetrate the grooves on the rough surface and leaves air gaps^4^. The Cassie-Baxter equation can be used to predict the probability of achieving superhydrophobic properties from intrinsically hydrophilic materials (contact angle < 90°) and can explain the self-cleaning properties of lotus leaves (Eq. 1)^1,4^

1 $$\mathrm{cos}{(\theta }^{*})=f\mathrm{cos}\theta +(1-f)\mathrm{cos}180^\circ =f\mathrm{cos}\theta +f-1,$$

$$f=\frac{\Sigma a}{\Sigma (a+b)}.$$

Derived from cellulose, which is the most abundant biopolymer on Earth, nanocellulose is a sustainable nanomaterial that has recently been investigated for a vast range of industrial and biomedical applications^14–19^. Cellulose nanocrystals (CNCs), which are extracted from the crystalline parts of cellulose fibers, have very interesting properties, such as transparency, very high strength and optical activity^19–21^. In addition, CNC films significantly decrease the permeability of gases at 0% relative humidity (RH)^19,22–25^. However, nanocellulose is highly sensitive to moisture, unstable in water, has no resistance against gas permeability at high relative moisture contents and cannot therefore be used in a damp environment^14–19^. This limitation significantly reduces the possible CNC applications targeting water resistance, such as the utilization of such materials in barriers and packaging. Thus, there is a strong demand for well-designed superhydrophobic CNC-based materials that have the potential to be applied in many applications (e.g., food packaging, coating, filtration membranes, etc.) since they would improve the inherent water sensitivity of cellulose^19,24,25^. Today fossil-based polymers such as polyethylene and alumina are currently used by the packaging industry to prevent moisture and air/oxygen permiability^19^.

Hydrophobic cellulose nanofibers (CNFs) can be prepared by utilizing techniques such as grafting, physical adsorption, and chemical vapor deposition and modifying agents such as fluorocarbons, silicones, and silicon-containing polymers^26–35^. Hydrophobic nanocellulose can also be used as a coating additive to create rough surfaces, therefore obtaining superhydrophobic material surfaces^26–28,30^. However, the use of toxic chemicals for nanocellulose modification, such as fluorocarbons, is not ideal, and more sustainable options are required. In addition, the cost for producing the superhydrophobic material needs to be considered. These considerations comprise the large prerequisite for designing sustainable and fossil- and fluorocarbon-free biobased materials for industrial applications such as food packaging. Therefore, there are currently intense ongoing research efforts in both academia and industry to find new, feasible methods for preparing superhydrophobic nanocellulose materials^26–35^.

The self-assembly of CNC suspensions to form a chiral nematic phase has been comprehensively studied and can create a film with a specific surface roughness^36–38^. In this context, Roman and Gray investigated the texture of CNC films formed by evaporation-induced self-assembly (EISA) using polarized optical microscopy (POM)^39^. They found that the CNCs inside the self-assembled tactoids are arranged in nematic layers, while those outside of this domain are substantially more disordered^39^. Moreover, the microstructure of the CNC film is based on the drying conditions and the thickness of the film. At certain film thicknesses, a parabolic focal conic texture, which is trapped in the film, was observed^39,40^. EISA is a slow method, and the concentration of the CNC suspension increases in a disordered manner. Another approach for the assembly of CNC films is vacuum-assisted self-assembly (VASA), which is faster than EISA, and the concentration step occurs directionally and orderly. VASA forms a gel gradually on top of the filter membrane and thereby decreases the water flow rate, which provides a significant time for the self-assembly of CNCs^41–43^. In addition, the CNC films made by VASA are flexible and easier to handle than those derived from the EISA method.

“Organocatalysis” refers to the acceleration of chemical reactions through the addition of a substoichiometric amount of an organic compound^44–47^. In the past couple decades, this catalysis strategy has gained increasing interest with respect to asymmetric catalysis and synthesis research due to its simplicity, high selectivity, compatibility with other types of catalysts and improvements in established chemical reactions^44–47^. In 2005, we reported the direct organocatalytic surface modification of cellulose fibers using naturally occurring chiral organic acids^48^. Since then, our group has expanded the use of organocatalysis for the preparation of hydrophobic cellulose, nanocellulose and nacre-inspired bionanocomposite materials and the strengthening of paper and nonwoven materials for the replacement of plastics^49–56^. However, in none of these studies the functionalized materials aquired superhydrophobicity. Thus, we need a novel approach for reaching the Cassie–Baxter state at the cellulose surface since the organocatalytic modifications are not enough for making the corresponding materials superhydrophobic.

As stated above, the nano- and microstructural roughness of the surface is very important for acquiring superhydrophobicity^1–10^. Bearing this in mind, we envisioned that combining self-assembly of CNC, which leads to the formation of hydrophilic films with interesting surface architectures at the micro- and nanoscales, with direct organocatalytic surface-functionalization could lead to a new design for creating superhydrophobic CNC films.

Herein, we disclose the corresponding bioinspired construction and design of transparent and functionalized CNC films with high WCAs (> 150°) and self-cleaning properties (the lotus effect) without the use of toxic fluorocarbon chemicals. It was found that the specific roughness of the CNC film was important for reaching the Cassie-Baxter stage (superhydrophobicity). Moreover, we demonstrated that the modified CNC films exhibit excellent water stability and that industrial relevant alkyl group functionalization decreased oxygen permeability at a high RH. In comparison, unmodified self-assembled CNC films disintegrate rapidly in water. Thus, the combined CNC self-assembly/organocatalytic modification strategy opens up for construction of sustainable CNC films with the above novel properties, which could be used in applications such as composites, packaging, barriers, textiles, security papers and optically active devices^19–21^.

## Results and discussion

### Construction of CNC films and organocatalytic surface modification

**Figure 1:**
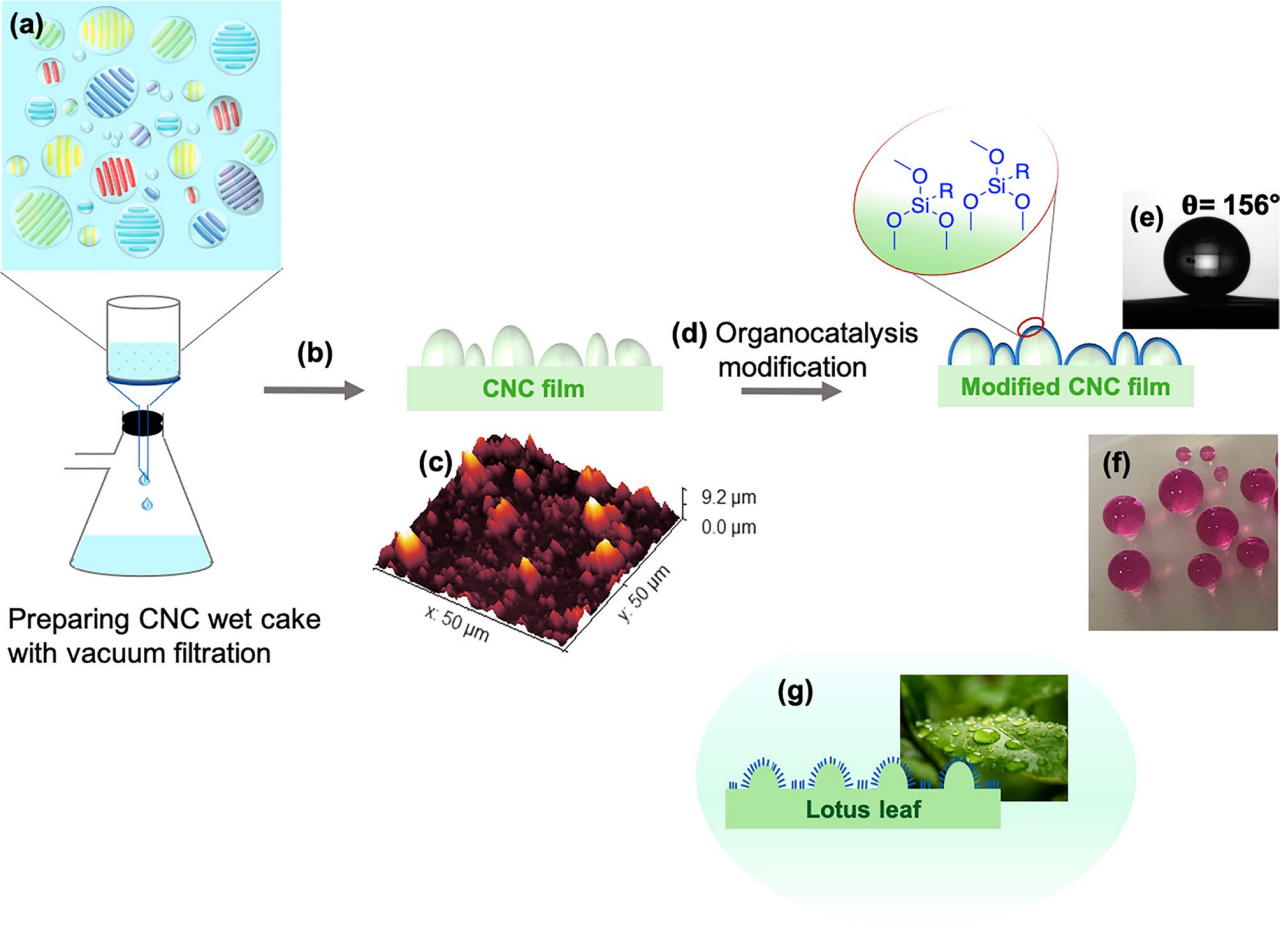
Construction of CNC surfaces by combined self-assembly and organocatalysis. (**a**) Model of the structure and distribution of tactoids in aqueous suspension of CNC. (**b**) Drying the CNC wet cake using Rapid-kothen sheet former. (**c**) AFM image of prepared CNC film with vacuum filtration, root mean square (RMS) roughness (Sq): 1.23 µm. (**d**) The organocatalytic reaction of CNC film with C16Si in the presence of l-tartaric acid, R: C16. (**e**) The contact angle of C16Si-CNC film. (**f**) The modified CNC film with C16Si, the drop of KMnO_4_ solution was added to dye the water. (**g**) Explanation of lotus effect.

Transparent CNC films were assembled by VASA, the model of structure and distribution of tactoids in aqueous CNC suspension is presented in (Fig. 1). The velocity of water depletion decreased as the CNC gel began to form on top of the filtration membrane, and the CNCs organized into a few scattered tactoids, which grew and fused during the concentration step. Next, the resulting wet cake was dried using Rapid-kothen sheet former to form a transparent and flexible CNC film with an ~ 40 µm thickness (Fig. 1b).

We acquired atomic force microscopy (AFM) images to investigate the topography of the CNC film, The pristine CNC film with a “rough” surface had an Sq of 1.23 µm (Fig. 1c), and the CNC film with a “smooth” surface had an Sq of 517 nm.

**Figure 2:**
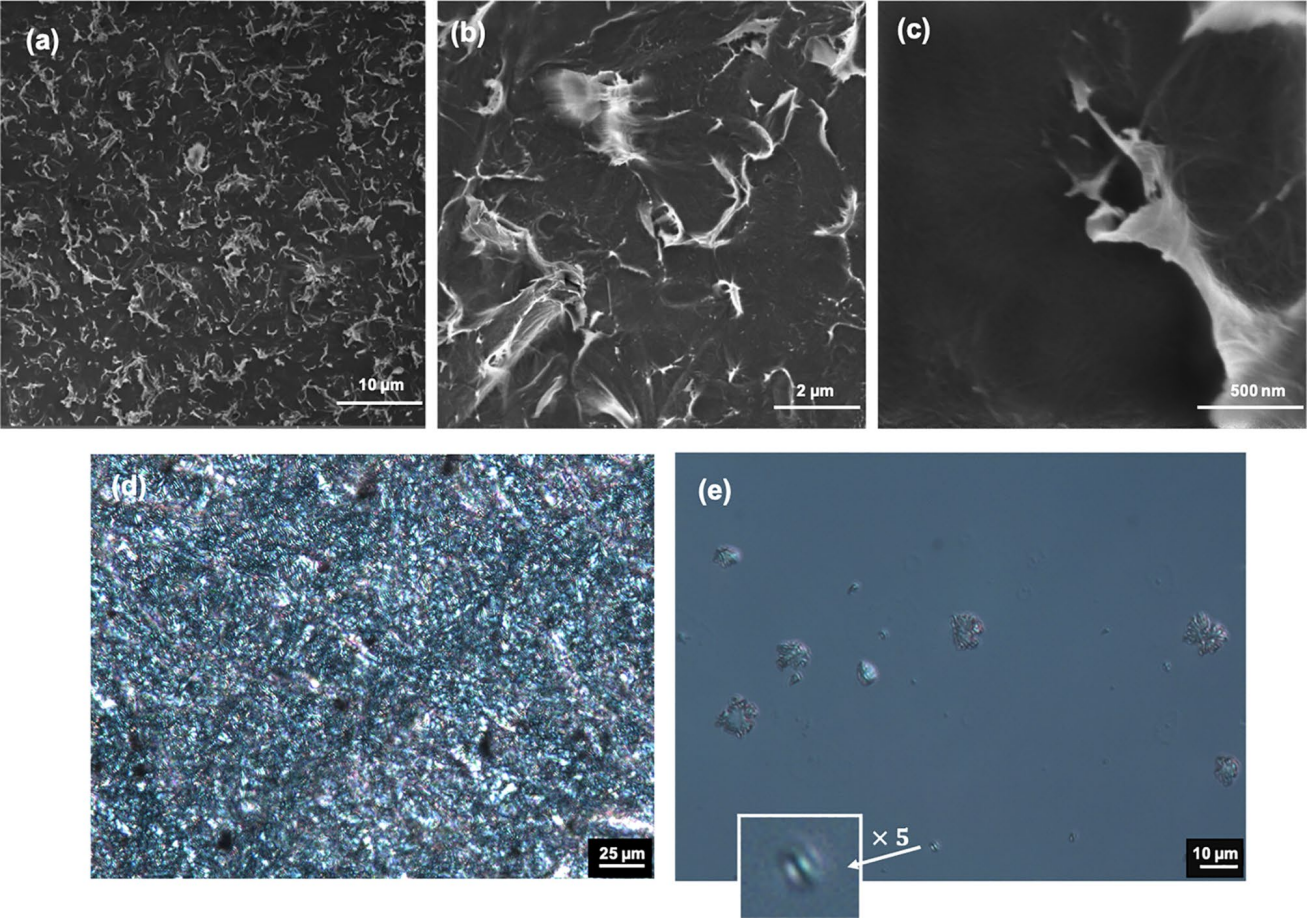
SEM and polarizing microscopy images of self-assembled CNC films. (**a**–**c**) SEM image of the CNC film surface, (**a**) scalebar: 10 µm, (**b**) scalebar: 2 µm, (**c**) scalebar: 500 nm. (**d**,**e**) POM of the CNC film, (**d**) scalebar: 25 µm, and (**e**) scalebar: 10 µm, the white arrow points out the magnified image of new born tactoid.

The scanning electron microscopy (SEM, Fig. 2a–c) and polarized light microscopy (POM) (Fig. 2d,e) were used to analyze the surface of CNC film. The SEM and POM images shows the contribution of self-assembled CNC particles to the rough surface (Fig. 2), and the magnified image shows the size of assembled CNC up to 1 µm (Fig. 2c).

The POM image reveals the creation of newborn tactoids, their growth, and aggregation (Fig. 2e). The arrangement of tactoids contribute to the surface roughness with a root mean square (RMS) roughness (S_q_) of ~ 1.23 µm. The SEM and POM images revealed that tactoids are formed and preserved during preparing CNC film (Fig. 2). We also obtained “smoother” film surfaces with an S_q_ of ~ 520 nm at the side of the film in contact with the filtration membrane. Thus, the VASA technique allowed for the assembly of “rougher” or “smoother” CNC film surfaces, and placing two films of the same type on top of each other will give the same roughness on both sides if desired. The WCAs for the “rough” and “smooth” film surfaces were 39° and 45°, respectively (Table 1).

**Table 1:** Organocatalytic silylation screening.


Entry^a^	Catalyst	Silane	Time(h)	Contact angle^b^	
				Film A (RMS: 1.23 µm)	Film B (RMS: 0.52 µm)
1	–	–	–	39	45
2	–		48	101	86
3	l-Tartaric acid		24	105	97
4	l-Tartaric acid		48	156	106
5	Citric acid		48	149	78
6	Acetic acid		48	125	99
7	–		48	97	83
8	l-Tartaric acid		48	153	102
9	Citric acid		48	158	106
10	l-Tartaric acid		48	156	120
11	Citric acid		48	> 165	123
12	Acetic acid		48	128	124

(a) CNC film (1 equiv.), silane derivatives (3 equiv.) and catalyst (5 mol%). See the “Methods” for details. (b) Recorded WCA after 5 min. (c) Root mean square roughness.

Next, we performed organocatalytic surface modification with a variety of alkoxysilanes (Fig. 1d, Tables 1 and 2). The initial organocatalytic silylation screening was performed using hexadecyltrimethoxysilane (C16Si), (3-mercaptopropyl) trimethoxysilane (TPSi) and allyltrimethoxysilane as reagents (Table 1). The screening revealed that when using L-tartaric acid and citric acid as the catalysts, a superhydrophobic CNC film surface was obtained when the film had a micrometer-scale Sq value. Thus, the right combination of organocatalyst and alkylsilane for modification of a film with an Sq of ~ 1.23 µm gave a WCA (> 150°, entries 4, 8–11, Fig. 1e,f) with a sliding angle of < 8° corresponding to a superhydrophobic surface. A WCA > 165° with a sliding angle less than 3° was achieved when allylsilane was used as the modifying agent for the organocatalytic reaction (entry 11, see SI for a movie where the drop rolls of instantly). In the case of the smoothest surface (S_q_ of ~ 520 nm), only a WCA of > 120° was obtained when allyl silane was used as the silylating agent (Table 1, entries 11 and 12). Thus, the surface roughness played a major role in the WCA, and at the lowest surface roughness value only a hydrophobic surface was created. However, the organocatalyst has an important contribution to obtaining the right surface architecture for acquiring superhydrophobic CNC films. The choice of citric acid or tartaric acid as the catalysts gives a successful result whereas selecting acetic acid only renders a hydrophobic surface.

**Table 2:** l-Tartaric acid-catalyzed silylation.


Entry^a^	Silane	Contact angle^b^	
		Film A (RMS^c^: 1.23 µm)	Film B (RMS: 0.52 µm)
1	–	39	45
2		156	106
3		151	94
4		153	102
5		156	120
6		129	116

(a) CNC film (1 equiv.), silane derivatives (3 equiv.) and l-tartaric acid (5 mol%). See the “Methods” for details. (b) Recorded WCA after 5 min. (c) Root mean square roughness.

Next, we performed the organocatalytic silylation reaction with a variety of alkoxysilanes as substrates using l-tartaric acid as the catalyst (Table 2). The choice of alkyl, allyl, and thiapropyl silanes resulted in a superhydrophobic CNC film for surfaces with an S_q_ of ~ 1.23 µm (entries 2–5), while films with a “smooth” surface (S_q_ ~ 520 nm) were hydrophobic. The choice of phenyltrimethoxysilane only provided hydrophobic CNC films (entry 6). Thus, the superhydrophobic CNC film can be created with different functional groups such as alkyl, olefin and thiol. This has important potential for upscaling since the alkyl group is used for hydrophobication of cellulose at an industrial multi-ton scalex^52^l The olefin and thiol group functionalities allow for introduction of click chemistry as well as metal binding (SH group) at the supehydrophobic CNC surface^50,54,55^.

### Water contact angle and atomic force microscopic measurements

**Figure 3:**
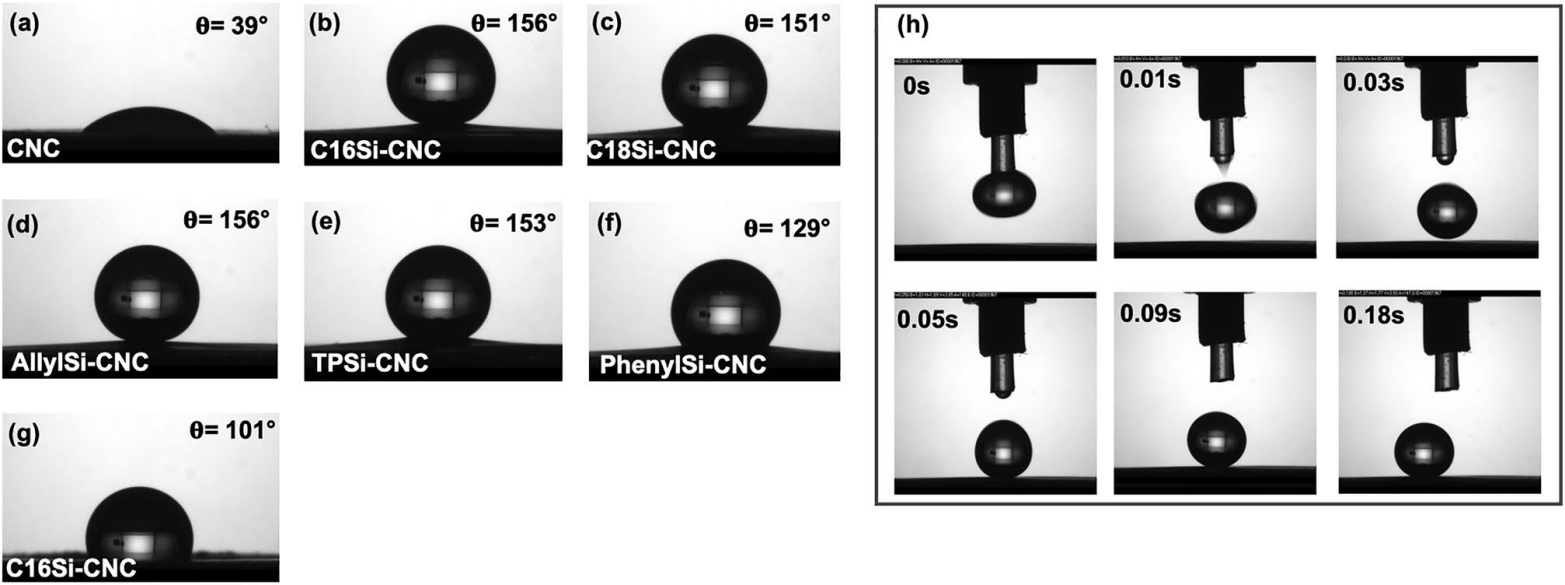
Water contact angle images of CNC films. (**a**) CNC film. (**b**–**f**) Organocatalytic reactions were performed with alkoxysilanes in the presence of l-tartaric acid as an organocatalyst. (**g**) The reaction was performed with C16Si in the absence of l-tartaric acid. (**h**)The movement of the water-drop contact angle measurements of AllylSi-CNC.

The successful modification of the CNC films was confirmed by WCA measurements and elemental analysis. For example, the WCAs of different CNC films are shown in Fig. 3. Photos were taken 5 min after dispensing the drop, and WCA measurements were performed to determine the water drop stability at the CNC surface. The values presented in Tables 1, 2, and Fig. 3 are the recorded contact angels after five minutes. At higher contact angles such as 156°, the drop starts to slightly move at the surface during the measurement (Fig. 3h, see also the movie when the drop immediately moves away at contact angles of > 165°). Elemental analysis of a sample of TPSi-CNC film determined the sulfur content to be 0.87 wt% (0.027 mmol), which was a ratio of 0.05 to glucose anhydride units (0.55 mmol). Thus, only surface modification had occurred.

**Figure 4:**
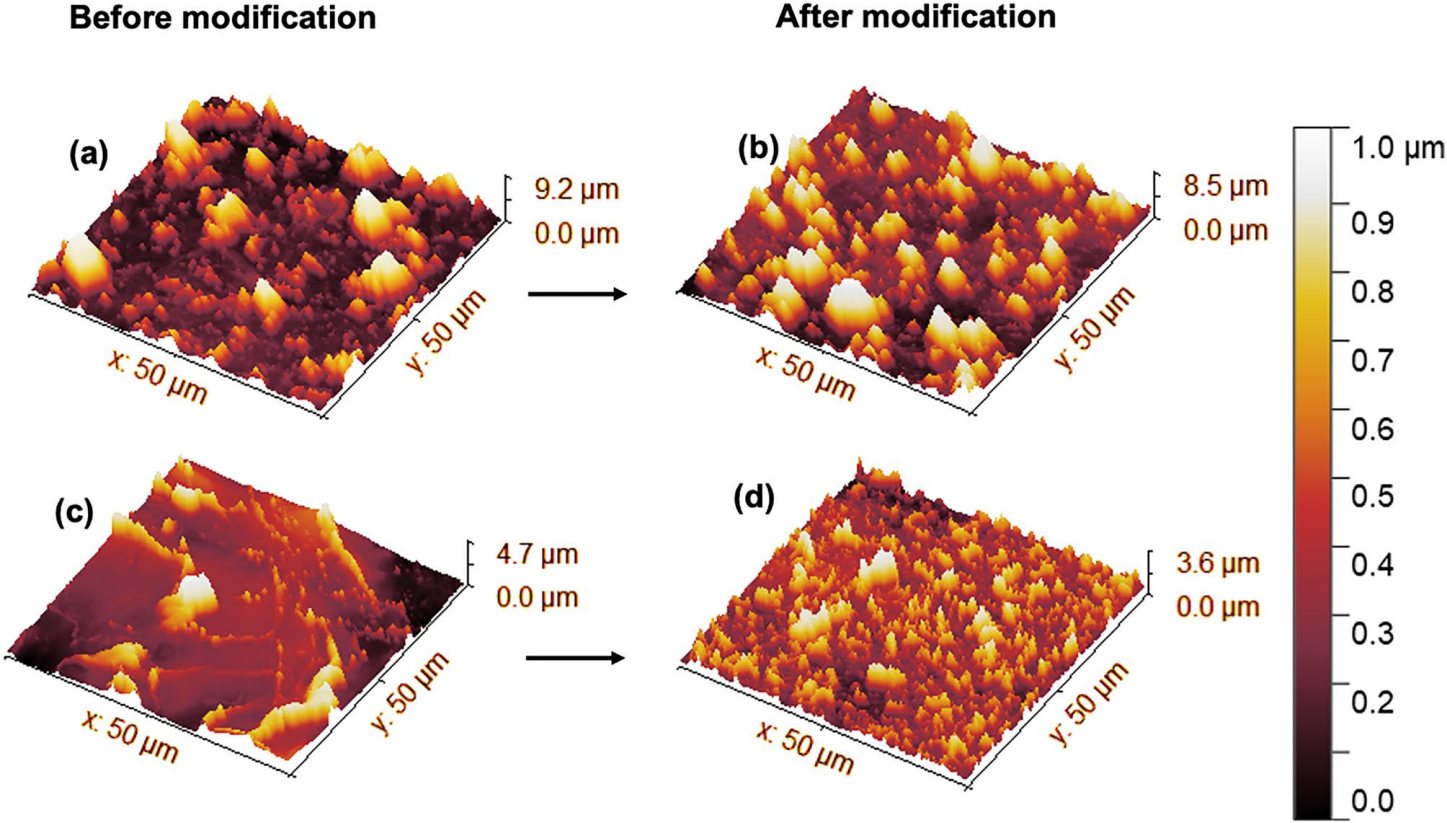
AFM images of CNC films. (**a**) CNC film, RMS roughness (Rq): 1.226 µm, surface area: 4519 µm^2^. (**b**) C18Si-CNC, Rq: 1.235 µm, surface area: 4887 µm^2^; (**c**) CNC film, (Rq): 517 nm, surface area: 2934 µm^2^. (**d**) C18Si-CNC, Rq: 375 nm, surface area: 3586 µm^2^.

We acquired large-area AFM images to investigate the topography of the pristine and modified C18Si-CNC film surfaces. The pristine CNC film with a “rough” surface had an S_q_ of 1.23 µm (Fig. 4a), and the CNC film with a “smooth” surface had an S_q_ of 517 nm (Fig. 4c). The AFM analyses of the silylated CNC films revealed uniform coverage of the modifying agent. We also observed that the S_q_ had the same range when comparing before (Fig. 4a,c) and after (Fig. 4b,d) organocatalytic surface modification. For the rough CNC film surfaces, the S_q_ value was similar at the microscale, and for the smoother surface, it was on the nanoscale and slightly decreased. Thus, organocatalysis provides mild and uniform surface hydrophobization to the CNC films without changing the surface roughness.

The combination of CNC self-assembly and organocatalytic surface modification can result in high WCAs (> 150°) when a microscale surface roughness is reached in combination with the right choice of catalyst and reagent. Thus, the Cassie–Baxter state is reached. Applying the Cassie–Baxter equation (Eq. 1), which is relatively applicable and common in nature^12^ we calculated how much of the CNC film surface was covered by air. A completely air-covered surface gave a contact angle of 180° (*f* = 0, where *f* is the ratio of the contact area of the liquid and surface in Eq. 1)^4^.

For example, the “rough” surface of the C16Si-CNC film had a measured θ* = 156°. The “smooth” C16Si-CNC film surface had a measured θ = 106°, which was assumed to be the Young’s contact angle. Then, we calculated the contact area of the film with water, *f* = 0.13. This calculated *f* shows that approximately 13% of the surface had contact with water and, consequently, 87% of the surface was covered by air, which the combination of roughness and surface chemistry prevented the wetting of the surface by water. Similar to structures in nature (e.g., lotus leaf)^12^, the hierarchical structure of the rough C16Si-CNC film reached the Cassie-Baxter state. Thus, the combined self-assembly/organocatalytic modification is a novel and successful route for making the CNC film surface superhydrophobic.

### Fourier transform infrared, water stability and oxygen permeability measurements

**Figure 5:**
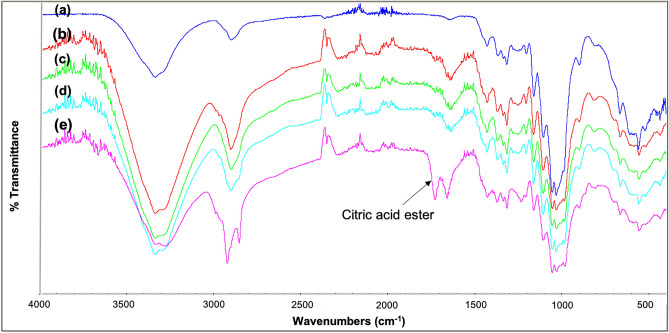
FTIR of modified CNC film using different organocatalyst in the reaction. (**a**) CNC. (**b**) C16Si-CNC, no catalyst. (**c**) C16Si-CNC, l-tartaric acid. (**d**) C16Si-CNC, acetic acid. (**e**) C16Si-CNC, citric acid.

We next performed FTIR analysis on the fabricated films (See supporting information). In Fig. 5, FTIR analyses of the C16Si-CNC films, which are modified with the industrial relevant C16 silane, are shown. In the case of using citric acid as the catalyst for the organocatalytic silylation, a clear ester carbonyl peak at 1725 cm^−1^ was observed (magenta line in Fig. 5). This peak was not observed when tartaric acid or acetic acid were used as the catalysts (Fig. 5c,d). Hence, citric acid catalyzed its own esterification to cellulose in parallel to the silylation reaction.

**Figure 6:**
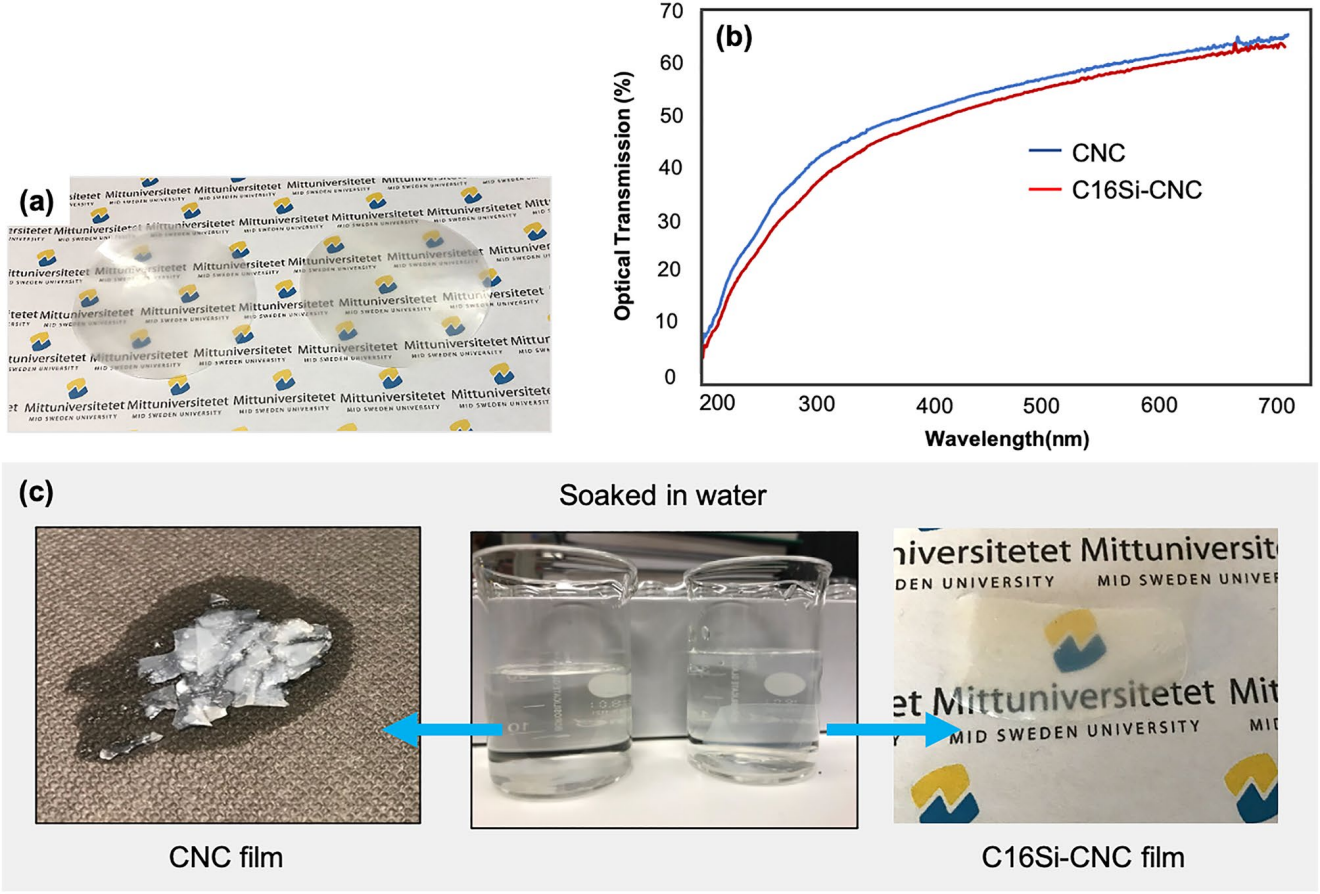
Transparency and water resistance properties of the CNC films. (**a**) Photograph of a CNC film (left) and the C16Si-CNC film (right). (**b**) Optical transmission of pristine CNC and C16Si-CNC. (**c**) Water-resistant C16Si-CNC, disintegration of soaked CNC film in the water after 2 h (left), and water resistance property of soaked C16Si-CNC in water after 2 weeks.

Next, the appearance of the films were investigated after modification in order to check if they maintain transparent and keep their optical properties. The photograph and UV–Vis spectra of pristine CNC film and modified one indicate that the transparency of the CNC film was preserved after modification (Fig. 6a,b). The water stability of the silane-modified CNC films was also examined. Thus, both a pristine CNC film and a C16Si-CNC film were soaked in distilled water (Fig. 6c, middle). After two hours, the pristine CNC film was totally disintegrated (Fig. 6c, left), while there was no change in the appearance of the C16Si-CNC film. In fact, the appearance and weight of the C16Si-CNC film did not change even after being placed in a water-filled beaker for an additional two weeks (Fig. 6c, right). These results demonstrate that organocatalytic modification makes the CNC film very stable in water. In addition, we did the same experiment in pH = 1 and 12, the result shows the great stability of modified CNC film in the acidic and alkaline condition as well.

Mechanical resistance of the modified CNC film was measured after abrading with sandpaper, the contact angle decreased from 156° to 150° when C16Si-CNC was gently rubbed with sandpaper (P320) two times. Rubbing the C16Si-CNC film strongly with the sand paper decreased the contact angle to 110°. Thus, silane modification had occurred at the CNC-film surface. The crystalline and high-order structure of nanocellulose decreases the oxygen permeability of the film^17,18^. However, at a relatively high RH, this property is lost^17^. Hence, we decided to probe the barrier properties of the CNC films since hydrophobization could decrease the oxygen permeability even at a high RH. The oxygen transmission rate (OTR) values were used to compare the relative oxygen barrier capabilities of packaging films. In industry, a material is considered a “high oxygen barrier” if its OTR is less than 1 cc m^−2^ day^−1^ at 0% RH. For example, polyethylene terephthalate (PET) and high-density polyethene (HPE) have values of 110 and 150–200 1 cc m^−2^ day^−1^ at 0% RH and 23 °C. To reach a value of < 1 cc m^−2^ day^−1^, the plastic can be covered with aluminum. To make the process more practical, we chose a relatively high RH for the measurements. The OTR values for unmodified CNC and C18Si-CNC films are given in Table 3. The OTR values for the pristine CNC film and C18Si-CNC film at 50% RH and 100% O_2_ were 2.485 and 0.291 cc m^−2^ day^−1^, respectively (entries 1 and 2). The OTR values for the pristine CNC film and the C16Si-CNC samples at 50% RH and 21% O_2_ (air) were 1.35 and 0.35 cc m^−2^ day^−1^, respectively (entries 5 and 6). Thus, the organocatalytically silane-modified CNC films significantly decreased the oxygen permeability and reached OTR values below 0.4 cc m^−2^ day^−1^ at 50% RH. Even at an RH as high as 90% and at 38 °C, a better barrier property was achieved compared to the pristine CNC film because the OTR dropped from 153.3 to 62.2 cc m^−2^ day^−1^ (entries 3 and 4). In comparison, PET has a value of 50 cc m^−2^ day^−1^ at 90% RH and 38 °C. The above measurements indicate the possibility of replacing plastics and even metals with organocatalytically modified CNC films.

**Table 3:** Oxygen permeabilities of CNC films.

Entry	Sample	Temperature (°C)	RH^a^ (%)	OTR^b^ (cc/m^2^/day)
1	CNC	23	50	2.485
2	C18Si-CNC	23	50	0.291
3	CNC	38	90	153.3
4	C18Si-CNC	38	90	62.2
5	CNC	23	50	1.35^c^
6	C16Si-CNC	23	50	0.35^c^

(a) Relative humidity. (b) OTRs of CNC films measured at 100% O_2_. The thicknesses of the films were ∼ 40 μm. (c) OTR measured at 21% O_2_.

## Conclusion

Superhydrophobic surfaces are very important and is intriguing for researchers both in academia and industry since they provide self-cleaning properties and water repellency. Cellulose nanocrystals, which have unique properties of high aspect ratio, high surface area, high mechanical strength (similar to steel), and a liquid crystalline nature, constitute a renewable nanomaterial with great potential for several uses (e.g., composites, films and barriers). However, their intrinsic hydrophilicity results in materials that are moisture sensitive and exhibit poor water stability. In conclusion, we have demonstrated a powerful sustainable method to obtain superhydrophobic CNC materials by combined self-assembly and organocatalytic direct surface functionalization. This novel approach allows for obtaining the right nanocellulose surface roughness at the microscale in combination with metal- and organofluorine-free catalytic modification at the nanoscale. The sustainable and superhydrophobic CNC films with different functional groups created by this strategy had a high WCA (> 150°) and self-cleaning properties (the lotus effect). Thus, no toxic and banned modification agents were required for reaching the Cassie-Baxter state. In addition, the designed CNC films exhibited excellent water stability and the alkyl group functionalization significantly decreased oxygen permeability at a high RH, which reached similar values to those of commonly used plastics^57^. Therefore, the disclosed results represent the future possibility for replacing organofluorine chemicals, fossil-based plastics and aluminia in packaging with CNC-based films in various applications. We are currently industrializing the disclosed sustainable approach of combined CNC self-assembly and organocatalytic modification as well as expanding it to other cellulose-based materials.

## Methods

### Materials

CNC (2–20 nm diameter, 20–500 nm length) suspensions (3% consistency) were kindly provided by Melodea Ltd. Chemicals and solvents were either purchased from commercial suppliers or purified by standard techniques. l-Tartaric acid and citric acid were dried in a desiccator over phosphorus pentoxide prior to use.

### Typical procedure for preparing CNC films by VASA

The CNC water suspension was used to prepare the CNC films. A total of 11.5 g of CNC suspension was diluted in Milli-Q water to reach a concentration of 0.05 wt%. This CNC solution was homogenized at 6000 rpm using an ULTRA TURRAX mixer (IKA T 25 digital) for 20 min. The well-dispersed CNC suspension was passed through a filtration system (with a fritted-glass filter support) using a membrane filter (DURAPORE 0.65 µm DVPP hydrophilic). After 10 min, the first layers of CNC gel started to form, and the velocity of water depletion was reduced. Next, the system was connected to vacuum, and filtration was continued. After 18 h, the wet cake on the filter membrane was covered with protective paper (RKP 220 Pappersrondeller, Paper Test Equipment AB) and transferred to a Rapid-Köthen sheet former. The wet CNC film was dried at 93 °C at an applied pressure of 96 kPa for 10 min. Next, a CNC film 8 cm in diameter and 40–45 µm in thickness was obtained.

### Typical procedure for the organocatalytic surface modification of CNC films

The freshly vacuum-assisted self-assembled CNC film (290 mg, 1.8 mmol, 1 equiv.) was placed in an oven-dried reaction vessel containing l-tartaric acid (40 mg, 0.27 mmol, 5 mol%), the silane derivative (3 equiv.), and dry toluene (50 mL). After performing the reaction for 48 h at 95 °C, the temperature was decreased to room temperature, and the reaction mixture was decanted. Next, the modified CNC film was washed with acetone (4 × 50 mL) and dried under reduced pressure. To avoid folding or deforming of the modified film, it was placed between two papers then, between two heavy plates and was kept overnight in a desiccator connected to the vacuum (see Supplementary Fig. S2).

### Fourier transformed infrared and UV–Vis spectroscopy

The FTIR spectra were recorded by Thermo Scientific NICOLET 6700 FTIR, Smart orbit, Diamond 30,000 − 200 cm^−1^. UV–Vis spectrophotometer, UV-1800 Shimadzu, was used to measure the Optical transmission of CNC films.

### Water contact angle measurements

The static WCAs were recorded on a DAT 1100-FIBRO-system ab-SWEDEN. The measurement method was TAPPI T 558 pm-95. The contact angle between the drop of water (4 μL) and the CNC film was measured at various time intervals following drop deposition and was determined by image analysis techniques on the captured images at the specified time. The reported WCAs here are based on the captured images after 300 s (5 min).

### Atomic force microscopy

AFM experiments were carried out with a Dimension Icon apparatus in tapping mode with Bruker TAP525A as a cantilever. The resonance frequency for these experiments was 445 kHz with a 0.32 lines/s scanning rate. The projected area of the samples was 50 µm × 50 µm (2500 µm^2^), and Gwyddion 2.59 software was used to analyze the images and measure the roughness.

### Scanning electron microscopy

SEM images of the sample surfaces were recorded using a Tescan Maya-2016, the samples were sputter-coated with Ir using Quorum, Q150T ES, and the thickness of the coating was 1 nm. The accelerating voltage was 2 kV.

### Polarized optical microscopy

POM was used to show how the chiral nematic order of the rod-like CNCs affected the surface texture of the CNC film. The POM image of the CNC film was recorded using Leica Microsystems: Leitz DMRX. For imaging, the sample was placed between two light microscope slides, and the interference contrast technique with an ICT 90 and a red wave plate was used.

### Oxygen transmission rate measurements

The OTRs of the samples were measured using Ox-Tran 2/21 (SL) instrument at a controlled RH, and ASTMF1927-07 was used as a standard test method. The exposure area of the samples was 5 cm^2^, and the gas flow was 10 mL/min. The measurement was carried out under two conditions: 50% RH and 23 °C and 90% RH and 38 °C.

## Supplementary Information

Supplementary Video 1.

Supplementary Information.

## Data Availability

The datasets used and/or analyzed during the current study available from the corresponding author on reasonable request.
