# Supplementary material for: Design and fabrication of superhydrophobic cellulose nanocrystal films by combination of self-assembly and organocatalysis
Source: Sci Rep. 2023 Feb 23;13:3157. doi: 10.1038/s41598-023-29905-1 (PMC9950148; doi:10.1038/s41598-023-29905-1)
Supplement: Supplementary file 2 — Supplementary Information. [file 41598_2023_29905_MOESM2_ESM.pdf]

Supporting Information

Design and Fabrication of Superhydrophobic Cellulose Nanocrystal Films by Combination of Self-Assembly and Organocatalysis

Rana Alimohammadzadeh[1], Italo Sanhueza[1], Armando Cordova[,1]*

[1] Department of Natural science and Technology, Mid Sweden University, Holmgatan 10, 851 70 Sundsvall, Sweden

Table of Content

General………………………………………………………………………………………..……….. 3
Preparing CNC film…………………………………………………………………………...………3
Figure S1. Preparing CNC film……………………………………………………………….…....3
Procedure for catalytic surface modification of CNC…………………….……………………….…....4
Figure S2. Drying CNC film after washing with acetone………………………………………….…....4
Contact angel measurement…………………………………………………………………….….4
Figure S3. Water contact angel of CNC film……………………………………………………....4
Figure S4. SEM images of CNC film……………………………………………………………...5
Figure S5. Polarizing microscope images of self-assembled CNC film……………………………...5
Figure S6. Polarizing microscope images of self-assembled CNC film……………………………...6
FT-IR data ….………………………………………………………………………………………...6
FT-IR spectra ……………………………………………………………………………………...7

General

Chemicals and solvent were used as purchased without any further purification. The Crystalline Nano Cellulose (CNC) water suspension (3% consistency) was provided by Melodea Ltd. L-tartaric acid and citric acid were dried in a desiccator over phosphorus pent oxide. Infrared spectra were recorded by Thermo Scientific NICOLET 6700 FT-IR, Smart orbit, Diamond 30000-200 cm^{-1}. Atomic force microscopy (AFM) experiments were carried out with Dimension icon and tapping mode with Bruker TAP525A as a cantilever. Resonance frequency for these experiments was 445 KHz with a 0.32 lines/s scanning rate. All SEM experiments were carried out at high voltage of 2 kv and low voltage 500v with Tescan Maya-2016 and Samples were sputtered with 1 nm Ir. The contact angle (CA) was recorded on DAT 1100-FIBRO-system ab-SWEDEN and the measurement method was TAPPI T 558 pm-95. Oxygen gas transmission rate (OTR) was measured on Ox-Tran 2/21 (SL), and the measurement method was ASTM F1927 – 07. IKA T 25 ULTRA TURRAX High Speed Homogenizer was used for homogenization of CNC water suspension. KONTAS Ultra-Ware micro filtration system and DURAPORE 0.65 μm DVPP hydrophilic filter paper was used for preparing film. RK sheet former was used for drying the wet film. The polarized microscopy (POM) images of the films between two glass slides were carried out with Leica Microsystems. The interference contrasts technique with ICT 90 with red wave plate was used.

Preparing CNC film

The CNC water-suspension (3% consistency, CNC: 2-20 nm in diameter and 20-500 nm in length) was used for CNC self-assembly to create CNC films for this study. A total of 11.5 g of CNC suspension in distilled water (0.05 wt.%) was homogenized at 6000 rpm using an ULTRA TURRAX mixer (IKA T 25 digital) for 20 minutes. Well-dispersed CNC suspension was passed through a filtration system (with fritted-glass filter support), DURAPORE 0.65 μm DVPP hydrophilic was used as membrane filter. After 10 minutes when the first layers of CNC film formed and the filtration became slow, the system was connected to the vacuum. After 18 hours, the wet cake on membrane filter was covered by another paper, and dried with Rapid-Köthen sheet former at 93°C at an applied pressure of 96 kPa for 10 minutes. This procedure, resulted in CNC film with 8 cm in diameter and 40-45 μm in thickness (Figure S1).

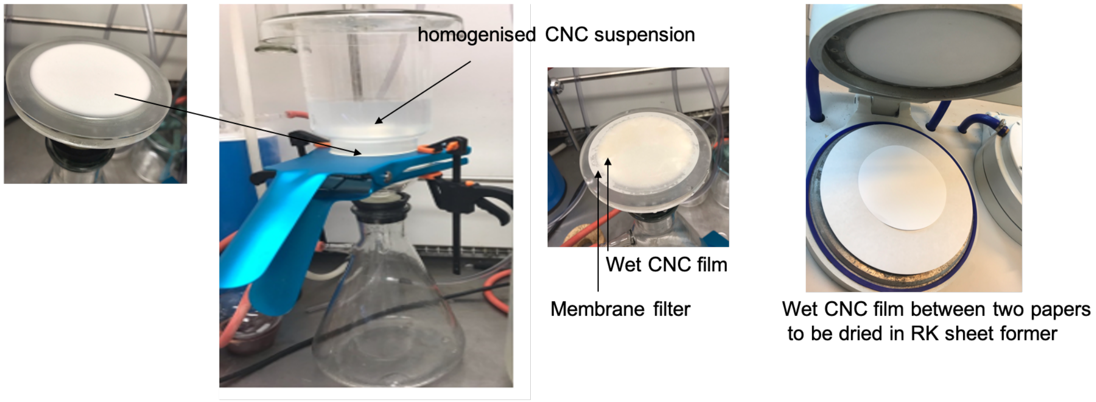

Figure S1. preparing CNC film.

Typical procedure for catalytic surface modification of CNC

A CNC film (290 mg, 1.8 mmol, 1equiv), was placed in an oven-dried reaction vessel containing L-tartaric acid (40 mg, 0.27 mmol, 5mol%), silane derivative (3 equivalents), and dry toluene (50 mL). After performing the reaction for 48 h at 95 °C, the temperature was decreased to room temperature and the toluene was decanted. Next, the modified CNC was washed with acetone (4 × 50 mL) and dried under reduced pressure. To avoid folding or deforming of modified film, the desiccator was used for drying and connected to the vacuum (Figure S2).

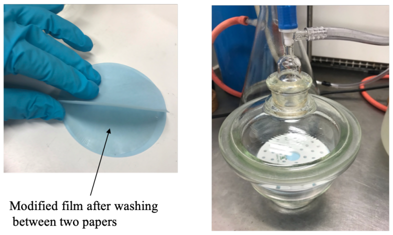

Figure S2. Drying CNC film after washing with acetone.

Contact angel measurement

The water contact angle was recorded on DAT 1100-FIBRO-system ab-SWEDEN, the contact angle between the drop of water (4μL) and CNC film at various time intervals following drop deposition are determined by image analysis techniques on the captured images at specified time.

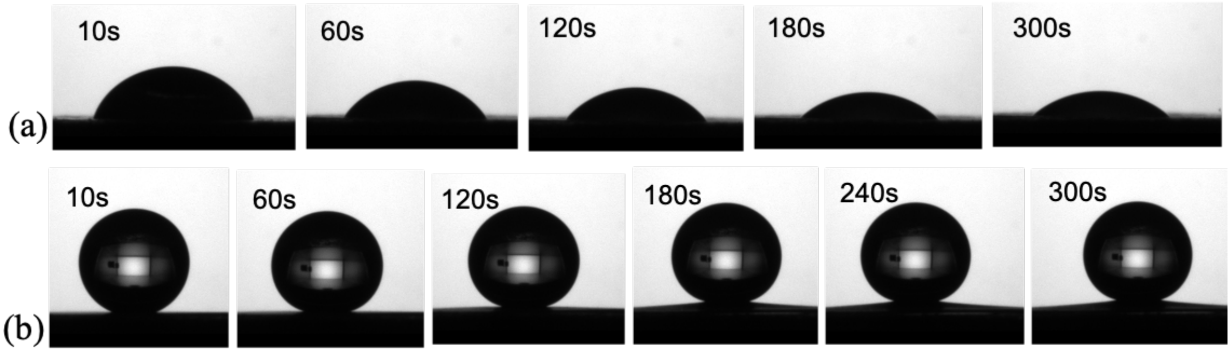

Figure S3. Water contact angel of CNC film. (a) CNC film, (b) TPSi- CNC film.

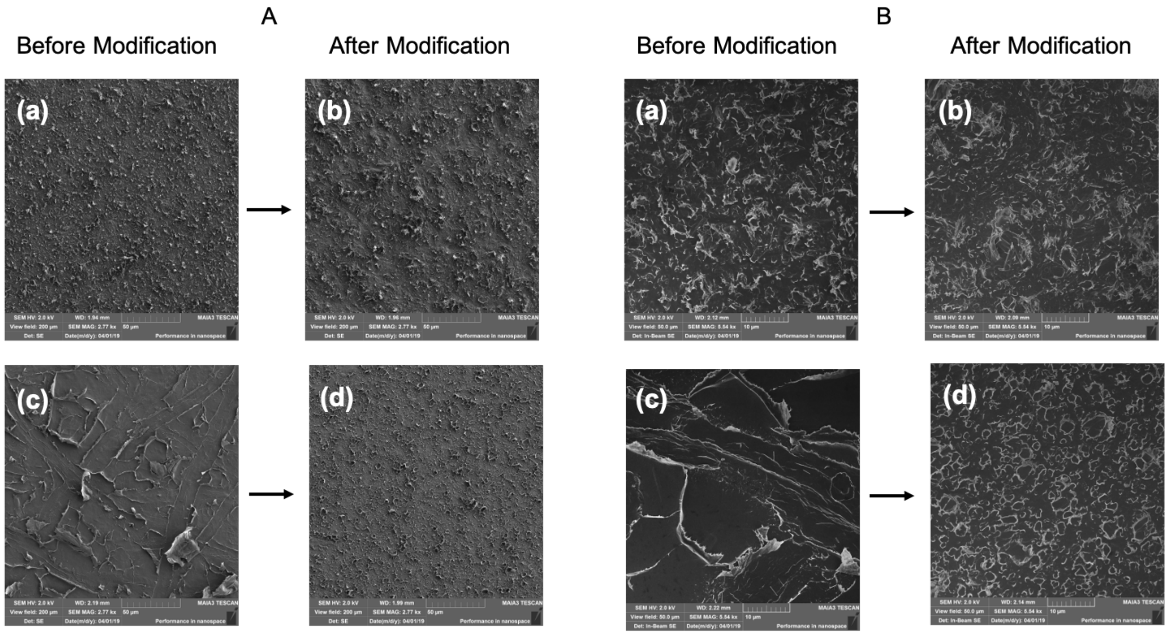

Figure S4. SEM images of CNC film, A: 2mm, B: 50µm; (a) CNC film with a rough surface (b) C18Si-CNC, (c) CNC film with a smooth surface, (d) C18Si-CNC.

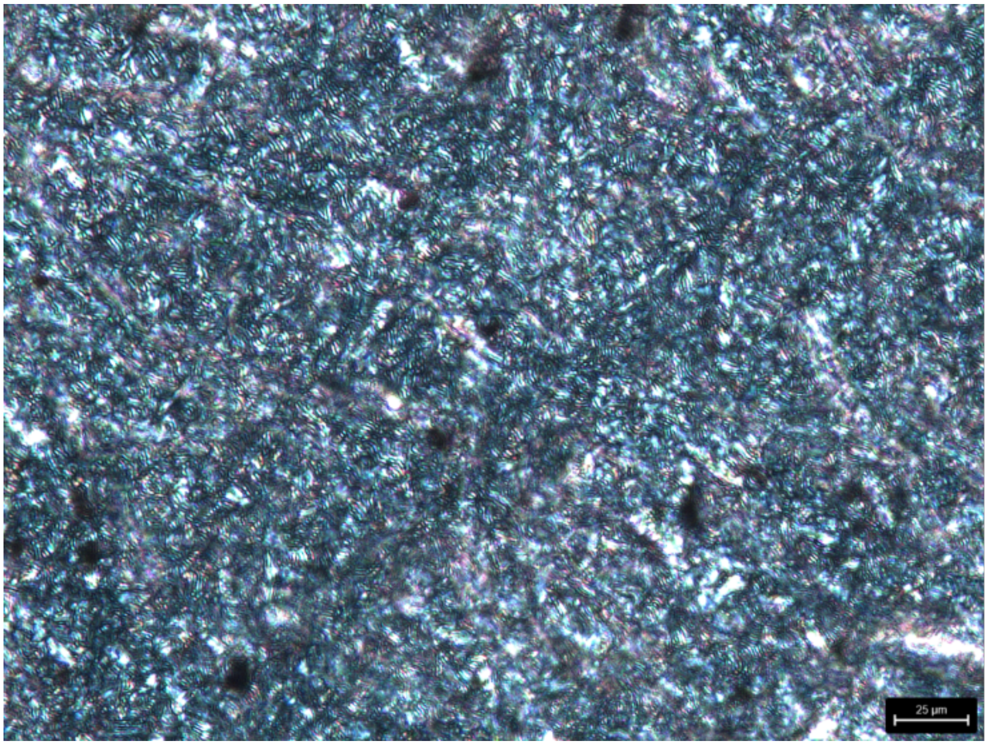

Figure S5. Polarizing microscope images of self-assembled CNC film, magnification: 20 and scalebar 25 µm.

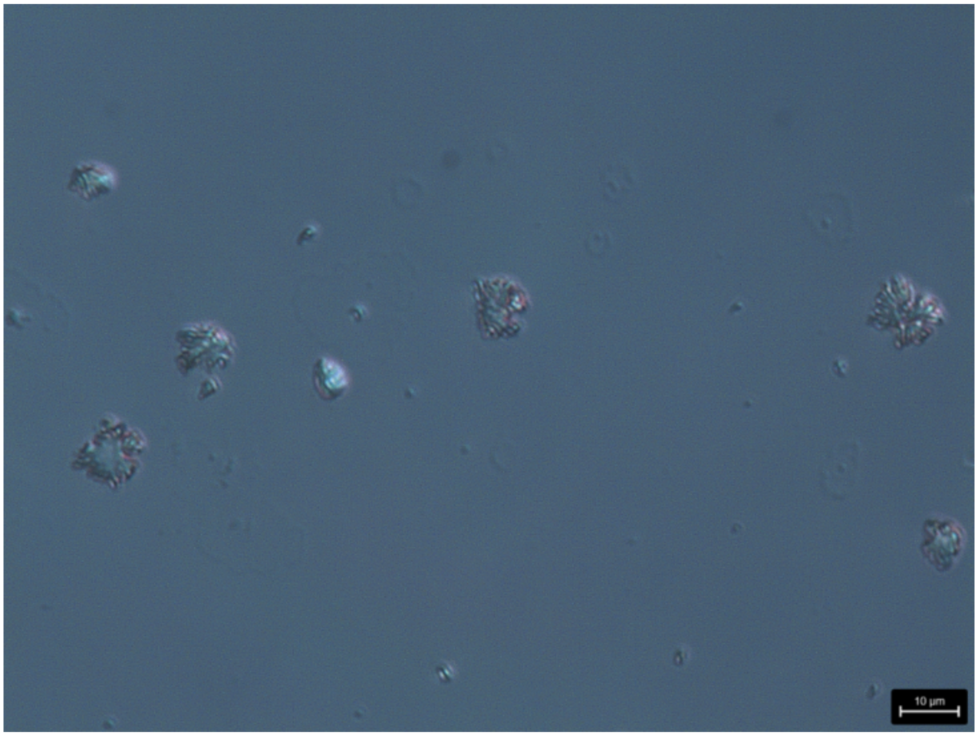

Figure S6. Polarizing microscope images of self-assembled CNC film, magnification: 40, scalebar 10 μm.

FT-IR data

CNC; FT-IR cm^{-1}: 3335, 2894, 1635, 1428, 1396, 1248, 1202, 1160,1105, 1054,1032, 898, 667, 559, 547, 449.

C16Si-CNC; FT-IR cm^{-1}: 3331, 2896, 1640, 1427, 1368,1314, 1248, 1204, 1104, 1052, 1029, 661, 555.

C18Si-CNC; FT-IR cm^{-1}: 3333, 2897, 1637, 1428, 1314, 1203, 1160, 1104, 1052, 1029, 808, 662, 556.

TPSi-CNC; FT-IR cm^{-1}: 3329, 2894, 1641, 1427, 1315, 1159, 1104, 1053, 1029, 896, 663, 558, 435.

AllylSi-CNC; FT-IR cm^{-1}: 3330, 2895, 1636, 1428, 1368, 1334, 1314, 1261, 1202, 1159, 1104, 1051, 1028, 983, 808, 661, 556.

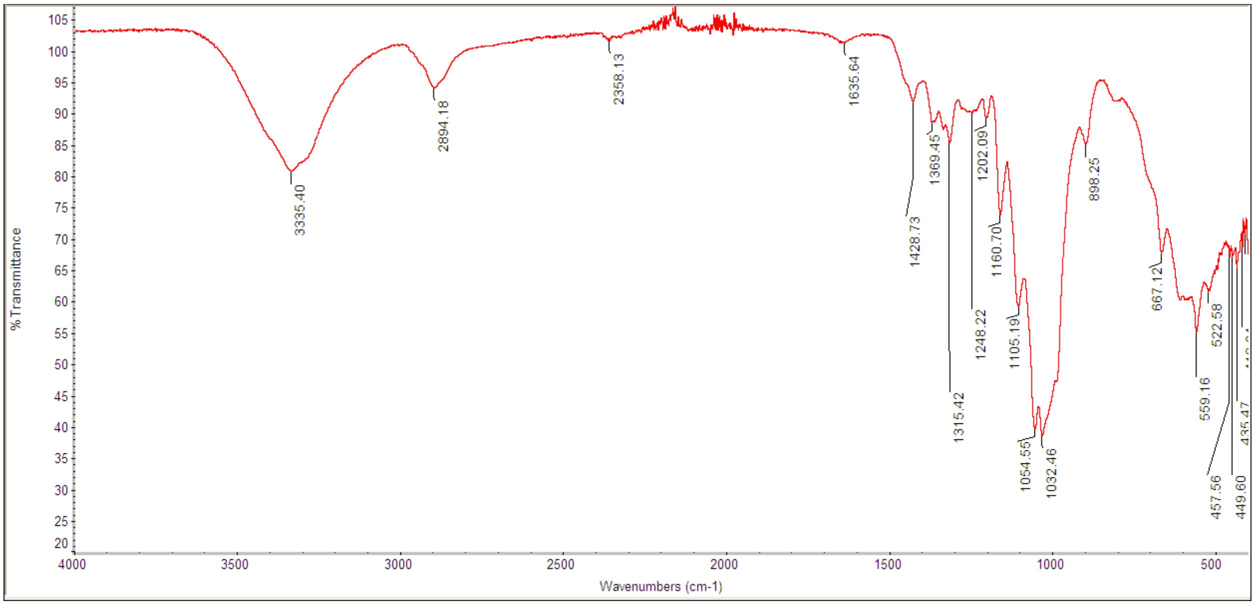

Figure S7. FT-IR of CNC.

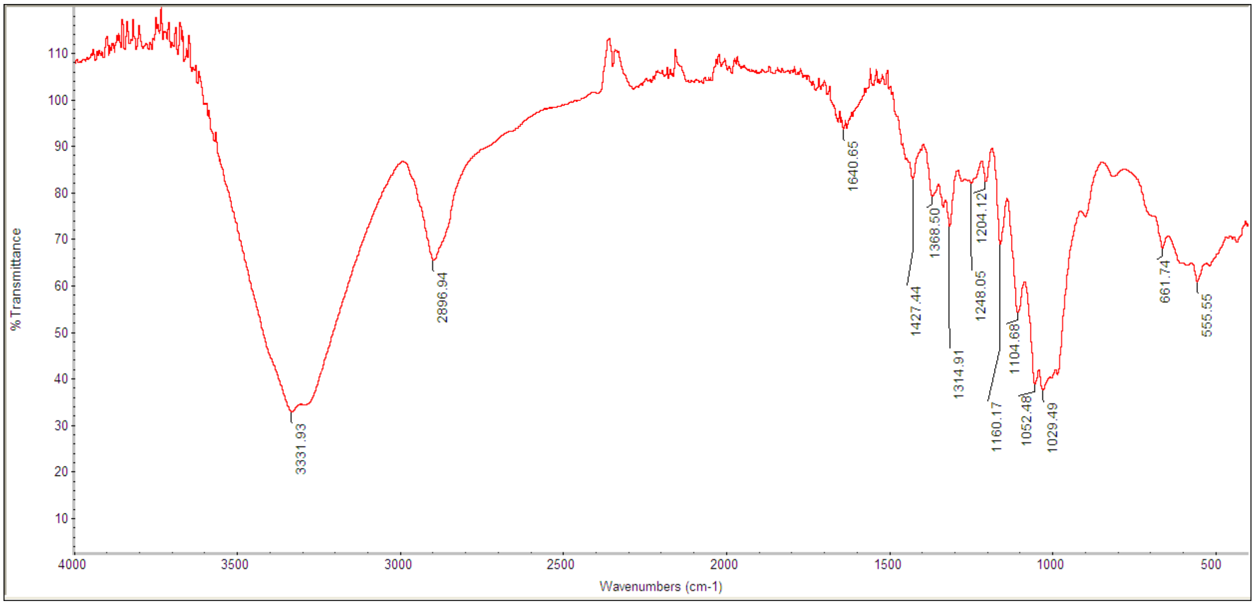

Figure S8. FT-IR of C16Si-CNC.

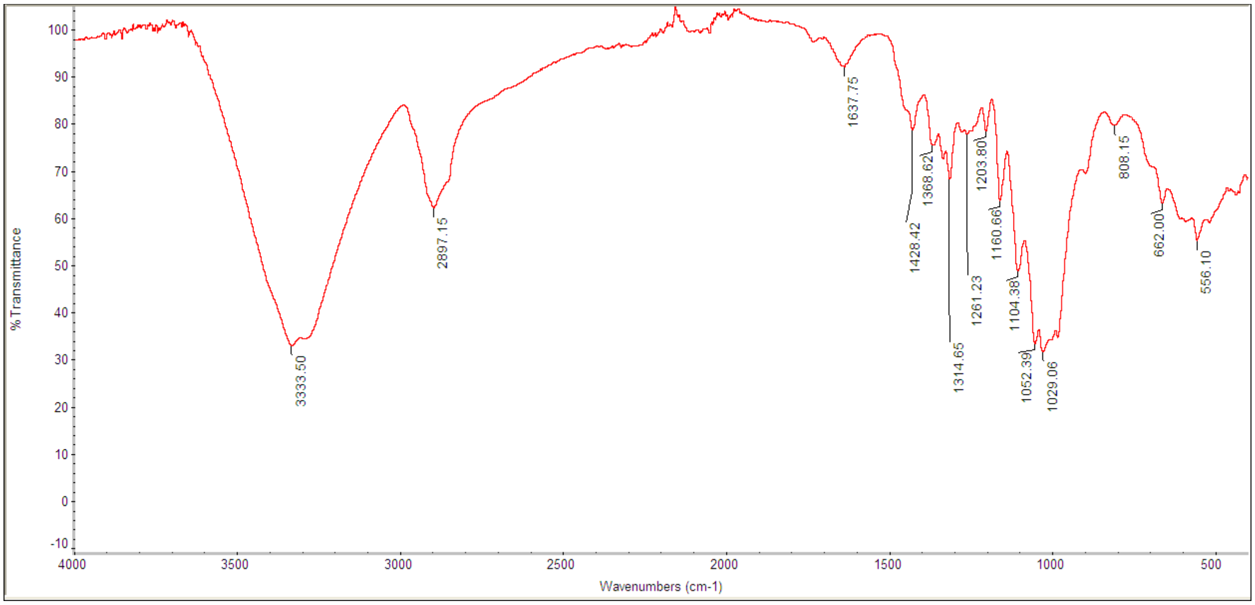

Figure S9. FT-IR of C18Si-CNC.

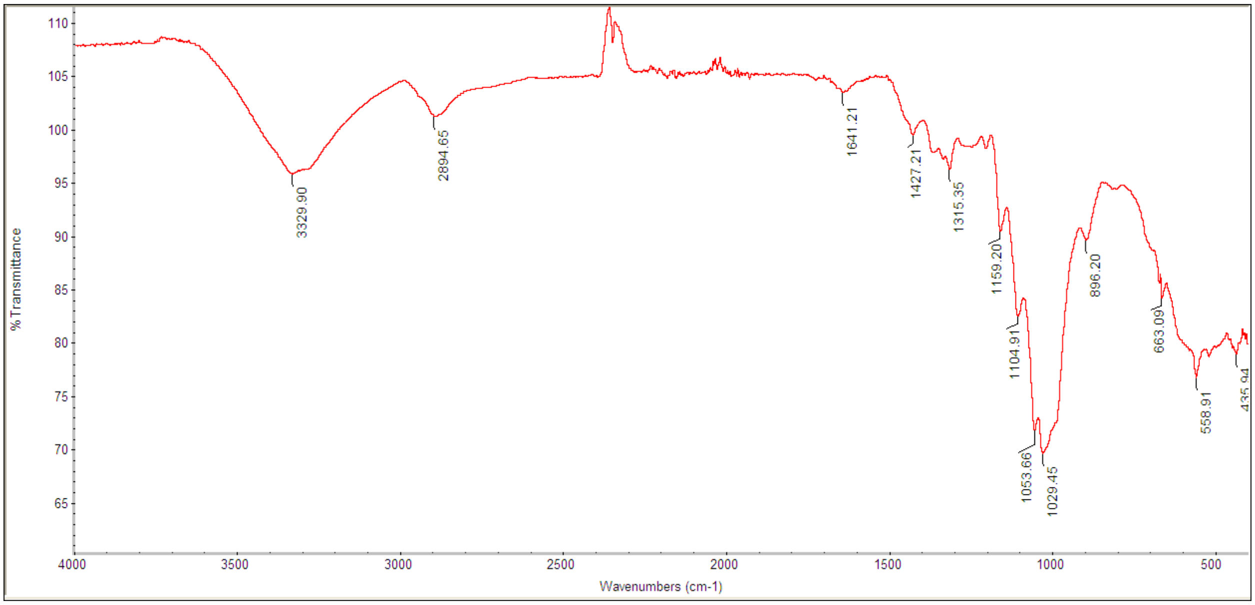

Figure S10. FT-IR of TPSi-CNC.

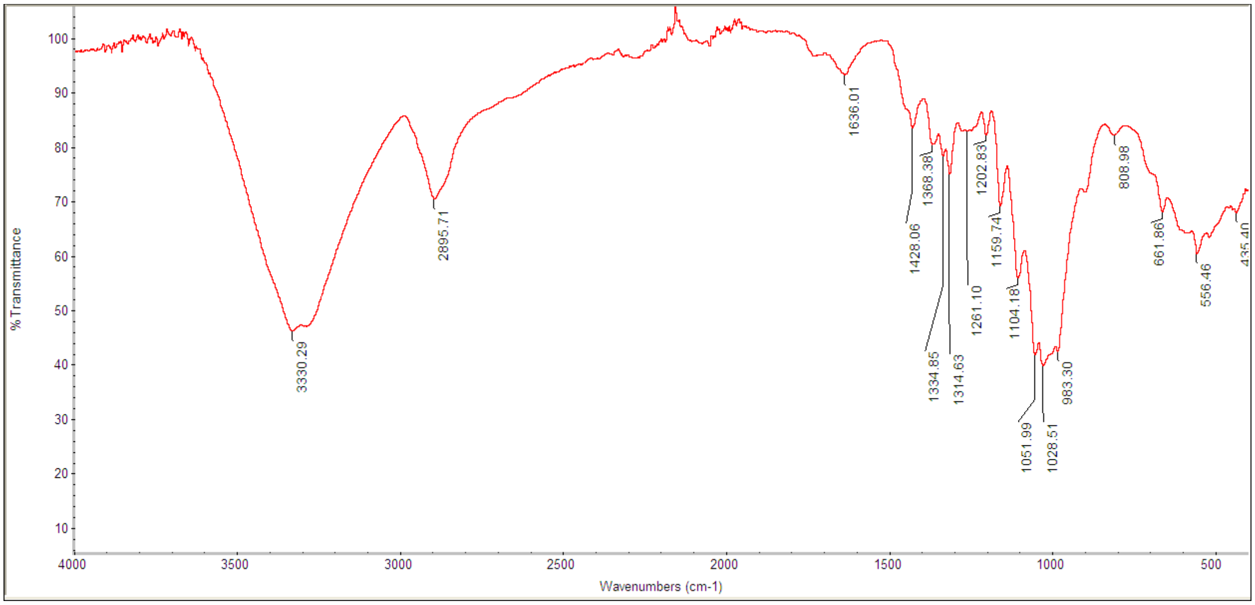

Figure S11. FT-IR of AllylSi-CNC.
